# The Impact of Monocyte to High-Density Lipoprotein Cholesterol Ratio on All-Cause and Cardiovascular Mortality in Patients with Transcatheter Aortic Valve Replacement

**DOI:** 10.3390/jpm13060989

**Published:** 2023-06-13

**Authors:** Denisa Bianca Mercean, Raluca Tomoaia, Adela Mihaela Şerban, Ştefan Dan Cezar Moţ, Radu Hagiu, Carmen Mihaela Mihu

**Affiliations:** 1 1st Department of Morphological Sciences, “Iuliu Haţieganu” University of Medicine and Pharmacy, 400012 Cluj-Napoca, Romania; denimercean@gmail.com (D.B.M.); carmenmihu@umfcluj.ro (C.M.M.); 2 Cardiology Department, Heart Institute “N. Stăncioiu”, 400001 Cluj-Napoca, Romania; adelamserban@yahoo.com (A.M.Ş.); motstefan@gmail.com (Ş.D.C.M.); radu_hagiu@yahoo.com (R.H.); 3 5th Department of Internal Medicine, “Iuliu Haţieganu” University of Medicine and Pharmacy, 400012 Cluj-Napoca, Romania; 4 Cardiology Department, Rehabilitation Hospital, 400347 Cluj-Napoca, Romania; 5 Radiology and Imaging Department, County Emergency Hospital, 400006 Cluj-Napoca, Romania

**Keywords:** monocyte-HDL cholesterol ratio, monocyte, HDL cholesterol, mortality, severe aortic stenosis, TAVR

## Abstract

Background: Inflammation plays a significant role in the pathogenesis of aortic stenosis. This study aimed to investigate the prognostic value of the monocyte-HDL cholesterol ratio (MHR), a new inflammatory marker, in severe aortic stenosis (AS) patients who underwent transcatheter aortic valve replacement (TAVR). Methods: A total of 125 patients with severe AS who underwent TAVR were assessed. Clinical, echocardiographic and laboratory data relevant to the research were retrospectively obtained from the patients’ records. The MHR was determined by dividing the absolute monocyte count by the HDL-C value. The primary endpoints were overall and cardiovascular mortality. Results: During a median follow-up time of 39 months, primary endpoints were developed in 51 (40.8%) patients (overall mortality) and 21 (16.8%) patients (cardiovascular mortality). A receiver operating characteristic (ROC) analysis showed that by using a cut-off level of 16.16, the MHR predicted the all-cause mortality with a sensitivity of 50.9% and specificity of 89.1%. In predicting cardiovascular mortality, the MHR exhibited a sensitivity of 80.9% and specificity of 70.1% when a cut-off level of 13.56 was used. In the multivariate analysis, the MHR (*p* < 0.0001; 95% CI: 1.06–1.15) and atrial fibrillation (*p* = 0.018; 95% CI: 1.11–3.38) were found to be significant predictors of overall mortality. Conclusions: This study showed a significant elevation in the MHR among patients who experienced all-cause and cardiovascular mortality and this ratio emerged as an independent predictor of all-cause death in patients with severe AS undergoing TAVR.

## 1. Introduction

Calcific aortic stenosis (AS) is the most common heart valve disorder in developed countries [1]. It is identified by the progressive remodeling and thickening of the aortic valve leaflets due to fibro-calcific changes. For a long time, calcific AS was considered to be a passive process of degeneration due to mechanical stress over the years. Recently, it has become clear that AS is instead the result of actively regulated and complex cellular mechanisms [2], where chronic inflammation plays a key role similar to the pathophysiology of coronary atherosclerosis [3].

The treatment of choice for high-risk patients affected by severe AS is transcatheter aortic valve replacement (TAVR). According to the last European guidelines for the management of valvular heart disease, TAVR is recommended in patients older than 75 years or unsuitable/high risk for surgical aortic valve replacement (SAVR) [1]. Even though it is a minimally invasive treatment, there may be immediate or long-term complications. Therefore, the ability to identify prognostic markers in patients receiving TAVR is crucial for risk classification and outcome prediction.

The monocyte-high-density lipoprotein cholesterol ratio (MHR), a relatively new marker of inflammation and oxidative stress, has been proposed as a prognostic factor in different cardiovascular diseases such as myocardial infarction [4], pulmonary embolism [5], atrial fibrillation [6] or hypertrophic cardiomyopathy [7]. In a recent study on patients with severe aortic stenosis undergoing TAVI, the MHR emerged as an independent predictor of all-cause mortality [8].

To the best of our knowledge, no study to date has evaluated the association of the MHR with post-TAVR cardiovascular-specific mortality. Thus, the aim of the present study was to investigate the prognostic role of the MHR in patients with severe AS undergoing TAVR in terms of overall and cardiovascular mortality.

## 2. Materials and Methods

### 2.1. Study Design and Patient Selection

This study entailed the retrospective observation of a series of patients who underwent transcatheter aortic valve replacements for severe aortic stenosis at the “Niculae Stancioiu” Heart Institute in Cluj-Napoca, Romania, between August 2017 and June 2020.

The diagnosis of severe aortic stenosis was established based on echocardiographic criteria and the decision to perform TAVR was established within the heart team based on the surgical risk scores (STS, EUROSCORE II) and the patients’ frailty. 

The exclusion criteria were missing data, other clinical conditions that could increase the plasma levels of inflammatory markers such as an active infection, known chronic inflammatory disease or cancer. Patients who were lost to follow-up were also excluded. 

The study protocol was approved by the ethics committee of the “Iuliu Hațieganu” University of Medicine and Pharmacy Cluj-Napoca, Romania (approval No. 66/10 March 2022) and the Heart Institute of Cluj-Napoca (No. 4539/12 April 2022) and was conducted in accordance with the Declaration of Helsinki. Informed consent was not required due to the retrospective design.

### 2.2. Data Collection

To describe the population under investigation, patient observation sheets were used to obtain demographic and clinical information, while the hospital’s electronic database was consulted for laboratory parameters. Echocardiographic studies were performed by experienced cardiologists, prior to and after the procedure. The monocyte-HDL cholesterol ratio was computed by dividing the quantity of peripheral blood monocytes with the serum HDL cholesterol level.

The primary endpoints were defined as the occurrence of cardiovascular death and all-cause death. Cardiovascular mortality referred to deaths caused by arrhythmias, myocardial infarction or acute heart failure. Overall mortality encompassed deaths from both cardiovascular conditions and other factors, including renal insufficiency, strokes, SARS-CoV-2 infection, pneumonia or pancreatitis.

### 2.3. Statistical Analysis

The statistical analysis was performed using MedCalc Statistical Software (MedCalc Software Ltd., Ostend, Belgium; http://www.medcalc.org, accessed on 3 April 2023). The normality was evaluated using the Kolmogorov-Smirnov test. For data that were normally distributed, the mean ± standard deviation (SD) and T-test were employed, whereas data that did not exhibit a normal distribution were described using the median and quartiles 1 and 3 (Q1; Q3) (IQR) and analyzed using the Mann-Whitney U test. Categorical data were presented as frequencies and percentages and analyzed using the chi-squared test. The patients were categorized according to the presence or absence of cardiovascular and overall mortality.

Any variables displaying statistically significant differences between groups (with a *p*-value < 0.05) were added to the univariate Cox regression. Variables exhibiting significant associations with mortality in the univariate Cox regression analysis were further included in a multivariable analysis. The ROC analysis was conducted using a bootstrap Youden index confidence interval, resulting in the determination of the optimal cut-point value and 95% CI for the Youden index. This approach aimed to enhance the prediction accuracy of overall and cardiovascular mortality for the MHR by maximizing sensitivity and specificity. The statistical significance was set at a *p*-value < 0.05. 

## 3. Results

**Figure 1:**
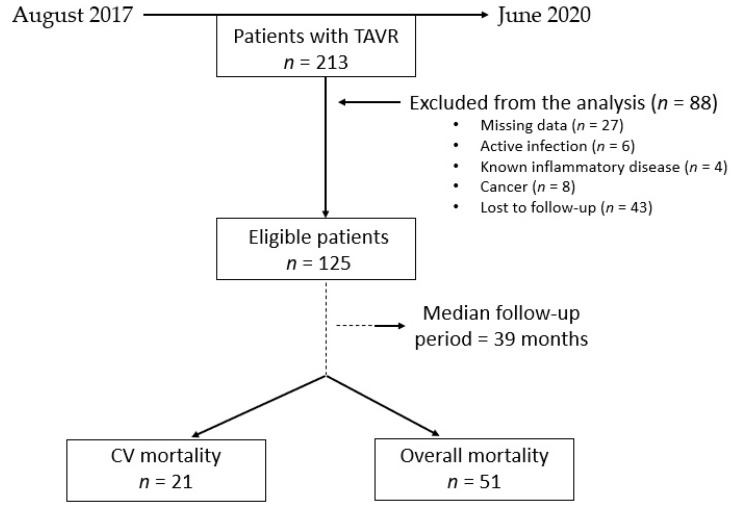
Flowchart of the population. CV: cardiovascular; TAVR: transcatheter aortic valve replacement.

Between August 2017 and June 2020, a total of 213 patients underwent transcatheter aortic valve replacements for severe aortic stenosis at the “Niculae Stancioiu” Heart Institute, Cluj-Napoca. After applying the exclusion criteria, a total of 125 patients were enrolled in the study (Figure 1).

After a median follow-up period of 39 months (with an interquartile range of 25–47 months), primary endpoints were developed in 21 (16.8%) patients in terms of cardiovascular mortality and in 51 (40.8%) patients related to overall mortality.

Table 1 summarizes the patients’ demographic characteristics, comorbid conditions, echocardiographic measurements and laboratory parameters based on the occurrence of primary endpoints. A total of 56 patients (44.8%) were male and the mean age of the study population was 77 ± 6.2 years. The most common chronic medical condition in the study cohort was hypertension (88%), followed by heart failure (73.6%). Coronary artery disease was significantly higher in patients who presented overall and cardiovascular mortality (*p* = 0.001 and *p* = 0.033, respectively). Moreover, a history of myocardial revascularization led to a higher cardiovascular mortality (*p* = 0.004). No other comorbid condition showed significant differences between groups.

In terms of laboratory parameters, patients with overall and cardiovascular death exhibited lower HDL cholesterol and higher levels of creatinine, C-reactive protein (CRP), erythrocyte sedimentation rate (ESR), white blood cells, monocyte counts and an increased MHR compared with those without a primary endpoint (*p* < 0.0001). Moreover, significantly higher levels of fibrinogen and neutrophils were found among patients with all-cause mortality.

The pre- and post-TAVR echocardiographic parameters were not significantly different between the groups (*p* > 0.05). No statistical significance was observed regarding the median length of hospital stay, nor regarding the post-procedural complications (permanent cardiostimulation, new left bundle branch block, vascular complications and strokes).

**Figure 2:**
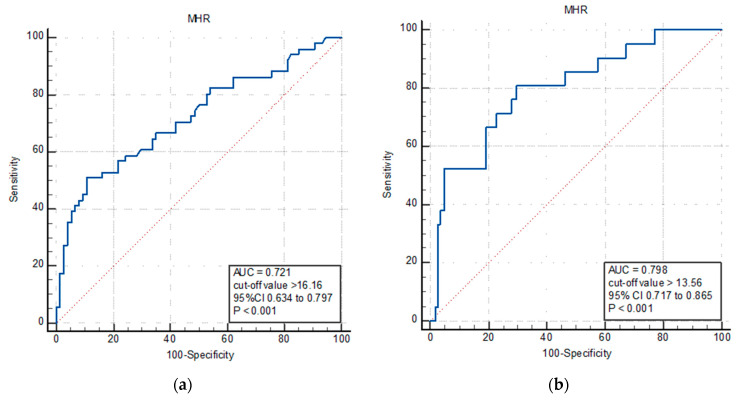
Receiver operating characteristic (ROC) curve analysis of MHR to predict (**a**) all-cause death and (**b**) cardiovascular death in patients with severe aortic stenosis undergoing TAVR. AUC: area under curve; MHR: monocyte-high-density lipoprotein cholesterol ratio.

The ROC curve analysis explored the discriminatory capability of the MHR for the overall (Figure 2a) and cardiovascular mortality (Figure 2b). For all-cause mortality, the area under the curve (AUC) was 0.721 (95% CI: 0.634–0.797; *p* < 0.001). Using a cut-off level of 16.16, the MHR predicted the all-cause mortality with a sensitivity of 50.9% and specificity of 89.1% (Figure 2a). For cardiovascular mortality, the AUC was 0.798 (95% CI: 0.717–0.865; *p* < 0.001). In predicting cardiovascular mortality, the MHR exhibited a sensitivity of 80.9% and specificity of 70.1% when a cut-off level of 13.56 was used (Figure 2b). Due to the relatively low number of patients who experienced negative outcomes during the first year following the procedure, the required sample size lacked the statistical power to detect significant events at the one-year follow-up.

**Figure 3:**
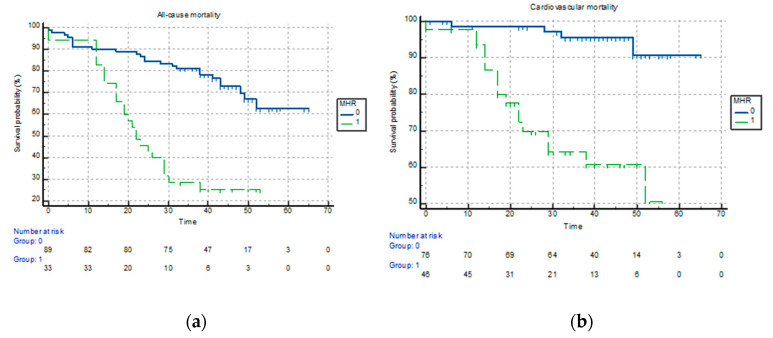
Kaplan-Meier analysis for (**a**) all-cause mortality according to an MHR of 16.16 and (**b**) cardiovascular mortality according to an MHR of 13.56 in patients with severe aortic stenosis undergoing TAVR.

The Kaplan-Meier survival analysis demonstrated that an MHR > 16.16 increased the probability of all-cause mortality by 6.6 times (95% CI: 3.3–13.2; *p* < 0.0001) (Figure 3a). Similarly, the probability of cardiovascular mortality was 9.2 times higher when the MHR > 13.56 (95% CI: 3.5–23; *p* < 0.0001) (Figure 3b).

The univariate Cox regression analysis showed that the MHR, monocyte count, HDL-C, WBC and the neutrophil count as well as coronary artery disease and atrial fibrillation were significantly associated with all-cause mortality (for all, *p* < 0.05) (Table 2). However, in the multivariate model, a high MHR (HR: 1.1; 95% CI: 1.06–1.15; *p* < 0001) along with atrial fibrillation (HR: 1.9; 95% CI: 1.11–3.38; *p* = 0.018) were the only significant predictors of overall mortality (Table 2).

## 4. Discussion

The primary outcomes of our study are summarized as follows: (1) We observed a significant elevation in the monocyte-HDL-C ratio among patients who experienced all-cause and cardiovascular mortality. (2) The MHR emerged as an independent predictor of all-cause mortality in patients who underwent TAVR.

The complex pathophysiology of degenerative AS involves a combination of inflammation, lipid infiltration and fibro-calcification processes [9]. Inflammation is one of the key mechanisms implicated in the development and progression of AS. It has been suggested that inflammatory cells, cytokines and growth factors contribute to the pathogenesis of aortic valve stenosis by promoting lipid deposition, extracellular matrix remodeling and calcification within the valve leaflets [3]. 

Inflammatory markers have been shown to play a significant role in the development of severe aortic stenosis and have been associated with adverse clinical outcomes in patients undergoing TAVR. Several studies found that elevated levels of pre-procedural CRP were associated with an increased risk of all-cause mortality and major adverse cardiovascular events following TAVR [10,11,12]. In our study, C-reactive protein also exhibited noteworthy connections, not only with overall mortality but also with cardiovascular mortality, providing compelling evidence of its involvement in these major adverse outcomes. Furthermore, our investigation uncovered a parallel association between the erythrocyte sedimentation rate, which serves as an additional recognized marker of inflammation, and both overall and cardiovascular mortality. In contrast, we found that fibrinogen was solely associated with overall mortality. In terms of more complex markers of inflammation, Mirna et al. showed plasma levels of tumor necrosis factor-alpha were independently associated with 12-month mortality in patients undergoing TAVR [13]. The MHR has emerged as a valuable addition to the current markers as it is easily accessible and reflects both inflammation and oxidation [14].

Monocytes have been shown to play a crucial role in the pathogenesis of inflammation and cardiovascular diseases. Monocytes are recruited to the site of vascular injury or inflammation and eventually differentiate into macrophages or dendritic cells, releasing proinflammatory cytokines such as tumor necrosis factor-alpha (TNF-α) and interleukin-1-beta (IL-1β) [15]. In addition, monocytes are known to produce reactive oxygen species (ROS), leading to oxidative stress [16] and matrix metalloproteinases (MMPs), known components that are implicated in aortic valve remodeling [17]. The quantity and properties of circulating monocytes and their subsets have become a topic of interest in studies on atherosclerosis and valvular disease, given that tissue macrophages originate from these circulating monocytes [18]. 

On the other hand, studies have shown that HDL cholesterol has the potential to hinder the development of atherosclerotic plaques and can have anti-inflammatory, antithrombotic and antioxidant effects on patients with cardiovascular disease [19]. Moreover, HDL-C has been shown to play a significant role in controlling monocyte activation, adhesiveness and inflammation [20]. As the proinflammatory and pro-oxidant impact of monocytes contrasts with the anti-inflammatory and antioxidant effect of HDL-C, it seems logical to consolidate these parameters into a single inflammatory marker, referred to as the MHR. In the present study, we found a substantial elevation in the monocyte count while concurrently noting a decrease in the levels of HDL-C level in patients with cardiovascular and overall mortality. Needless to say, the MHR was higher in both endpoint groups, underscoring the potential significance of this ratio as a predictive marker for adverse outcomes.

Several studies have shown that a raised MHR serves as a significant prognostic marker across a range of cardiovascular diseases, including ST elevation myocardial infarction [4], pulmonary embolism [5], bicuspid aortic valve degeneration [21], rheumatic mitral valve stenosis [18] and atrial fibrillation [6]. These findings emphasize the importance of the MHR in relation to inflammation as it has a crucial role in the occurrence of significant cardiovascular events.

In the literature, an elevated baseline neutrophil-lymphocyte ratio (NLR) was independently associated with increased subsequent mortality and rehospitalization after TAVR or SAVR [22]. In our study, the NLR was not found to be a significant prognostic indicator for all-cause or cardiovascular mortality. 

The only study on the prognostic role of the MHR in patients undergoing TAVR included 145 subjects [8]. The authors found that the MHR and the presence of a cerebrovascular accident were independent predictors of all-cause mortality. However, their study may have been biased by the presence of coronary artery disease (CAD) in a large majority of the participants (80.7%) as CAD represents a known pathology where the MHR has a well-established implication [4,23,24]. Additionally, CAD was not included in their univariate and multivariate analysis, which may have influenced their conclusion that the MHR was a strong independent predictor of mortality. As the authors themselves concluded, the strong independent predictive power of the MHR possibly stems from underlying CAD. In our study, 45 (36%) of the subjects had known CAD and 34 of them benefited from myocardial revascularization. Similar to other studies, we found an association between CAD and cardiovascular (*p* = 0.001) and overall mortality (*p* = 0.033). In the univariate Cox analysis, CAD was associated with overall mortality (HR: 1.76; 95% CI: 1–3.09; *p* = 0.048) but was outperformed by the MHR in the multivariate analysis. One possible explanation could be the low incidence of CAD observed in the population under investigation. The only parameter other than the MHR that emerged as an independent predictor of all-cause mortality was the presence of atrial fibrillation (HR: 1.9; 95% CI: 1.11–3.38; *p* = 0.018), a comorbid condition that has also been associated with high mortality rates after TAVR in other studies [25,26,27]. Atrial fibrillation is associated with an increased risk of strokes, heart failure and mortality, particularly in patients with cardiovascular disease (CVD). AF is estimated to be responsible for up to 20% of all strokes and is associated with a two-fold increase in the risk of cardiovascular mortality in patients with CVD [28]. No significant associations were observed between the primary endpoints and the additional comorbidities presented by our study population, including hypertension, obesity, hyperlipidaemias, diabetes mellitus, strokes and heart failure. 

Jneid et al. conducted a study on 189 patients with severe aortic stenosis who underwent TAVR and demonstrated that the baseline creatinine level was independently associated with both overall and cardiac-related mortality [29]. In our study, the pre-operative creatinine level was also significantly associated with higher rates of all-cause and cardiovascular mortality.

Furthermore, we systematically collected echocardiographic parameters, both prior to (left ventricular ejection fraction, pulmonary systolic arterial pressure, aortic valve maximum velocity and gradient, aortic regurgitation and mitral regurgitation) and following the TAVR procedure (prosthetic valve maximum velocity and gradient, presence of periprosthetic leak and grade of regurgitation). However, our analysis revealed that none of these parameters exhibited a discernible prognostic role in predicting cardiovascular and overall mortality. Al-Akchar et al. studied the role of standard echocardiographic parameters in predicting the outcomes and the one-year mortality of 399 patients who underwent TAVR; among them all, a left ventricular ejection fraction less than 35% was the only independent predictor of one-year mortality [30]. The limited number of patients with a left ventricular ejection fraction below 35% (specifically, only 13 out of 125 individuals) was the primary factor contributing to the absence of such findings in our study. 

The presence of paravalvular aortic regurgitation has a detrimental impact on prognosis following transcatheter aortic valve replacements. Patients with a regurgitation severity level surpassing mild are faced with a substantial escalation in both morbidity and mortality rates [31]. As anticipated, the presence of a periprosthetic leak did not demonstrate any meaningful implications for the endpoints in our study. This was primarily because the degree of regurgitation generated was predominantly mild to moderate without a hemodynamic impact.

When it comes to the prosthesis type, the CHOICE randomized clinical trial conducted a comprehensive comparison of device performances between balloon-expandable transcatheter heart valves and self-expanding transcatheter heart valves in 241 high-risk patients with severe symptomatic aortic stenosis, with a follow-up period of five years [32]. The outcomes of the trial revealed that five years after a transcatheter aortic valve replacement using early-generation balloon-expandable and self-expanding valves, there were no significant differences observed in terms of mortality rates, stroke occurrence and rehospitalization rates for heart failure [32]. These findings suggest comparable long-term outcomes between the two types of valves in the context of TAVR procedures. Within our follow-up period, our results also showed no differences between the types of prosthesis used in terms of overall and cardiovascular mortality rates.

The present study was novel in that it examined the influence of the monocyte-HDL-C ratio on mortality, encompassing not only overall mortality, but also focusing on mortality caused by cardiovascular factors. Furthermore, we evaluated perioperative complications. Patients who experienced a fatal cardiovascular event had a higher baseline MHR (18.2 ± 5.5 vs. 12.1 ± 5.2; *p* < 0.0001). However, as the occurrence of this primary event was rare (16.8%), it was not possible to establish the predictive independence of the MHR. In addition, as the clinical status of these patients might vary over time, we acknowledge that a time-dependent ROC curve may be valuable in the detection of events. However, few participants in our study had adverse effects earlier than twelve months after the procedure. We can, therefore, assume that the majority of patients experienced any adverse outcomes more than a year after the procedure.

Despite a limited understanding of its mechanisms, it is worth noting that the MHR may have the potential to predict all-cause and cardiovascular mortality in severe aortic stenosis patients undergoing TAVR in routine clinical practice. Nonetheless, further comprehensive research is needed to validate these findings on a larger scale.

## 5. Limitations

Our study had certain limitations that need to be acknowledged. First, the patients enrolled in this study came from a single center and the study had a retrospective design. In addition, it was conducted on a relatively small sample size. Moreover, the prognostic significance of potential fluctuations in the MHR over time remained unclear as our evaluation of the MHR levels was limited to the initial assessment only. Due to the small number of patients, we were also unable to perform both univariate and multivariate Cox regression analyses for the endpoint of cardiovascular mortality. Major nonfatal cardiovascular events were not included because of the low data quality and missing information in this regard.

## 6. Conclusions

The findings of our study indicated that the MHR may serve as a prognostic marker for patients with severe aortic stenosis who undergo TAVR, with regard to both overall mortality and cardiovascular mortality. The ease of use, cost-effectiveness and widespread availability of this index make it a convenient tool for clinical practice. Additional research on a larger scale is necessary to confirm our findings and establish the prognostic usefulness of the MHR as a pre-operative risk-assessment tool for TAVR candidates.

## Figures and Tables

**Table 1:** Demographic characteristics, comorbid conditions, echocardiographic measurements and laboratory parameters of the patients according to the presence of primary endpoints.

Parameter	Total Patients n = 125	Cardiovascular Mortality			Overall Mortality		
		(+) n = 21	(-) n = 104	*p*-Value	(+) n = 51	(-) n = 74	*p*-Value
Gender, male	56 (44.8%)	12 (57.1%)	44 (42.3%)	0.212	28 (39.2%)	28 (37.8%)	0.059
Age, years	77 ± 6.2	75.76 ± 6.11	77.36 ± 6.21	0.281	78.1 ± 5.8	76.3 ± 6.3	0.119
**Comorbidities**							
Hypertension	110 (88%)	19 (90.4%)	91 (87.5%)	0.703	45 (88.2%)	65 (87.8%)	0.946
Obesity	16 (12.8%)	4 (19%)	12 (11.5%)	0.349	7 (13.7%)	9 (12.1%)	0.797
Hyperlipidaemia	49 (39.2%)	6 (28.5%)	43 (41.3%)	0.276	21 (41.1%)	28 (37.8%)	0.708
Diabetes mellitus	39 (31.2%)	9 (42.8%)	30 (28.8%)	0.208	19 (37.2%)	20 (27%)	0.227
Coronary artery disease	45 (36%)	14 (66.6%)	31 (29.8%)	**0.001**	24 (47%)	21 (28.3%)	**0.033**
History of myocardial revascularization	34 (27.2%)	11 (52.3%)	23 (22.1%)	**0.004**	17 (33.3%)	17 (22.9%)	0.2
Atrial fibrillation	48 (38.4%)	11 (52.3%)	37 (35.5%)	0.15	27 (52.9%)	21 (28.3%)	**0.005**
Stroke	14 (11.2%)	3 (14.2%)	11 (10.5%)	0.624	8 (15.6%)	6 (8.1%)	0.188
Heart failure (NYHA class III or IV)	92 (73.6%)	15 (71.4%)	77 (74%)	0.805	40 (78.4%)	52 (70.2%)	0.31
**Hospitalization period, days**	10 (7–13)	11 (7.7–13.2)	10 (7–12.5)	0.351	11 (8–13.7)	10 (7–12)	0.176
**Echocardiographic measurements**							
LVEF	49.4 ± 11.7	48.4 ± 11.4	49.6 ± 11.9	0.68	48.2 ± 12.7	50.2 ± 11.1	0.349
PSAP	42 (37.7–55)	49 (38.7–56)	40 (37.5–55)	0.487	45 (40–55)	40 (37–55)	0.404
Aortic valve Vmax (m/s)	4.4 (4.1–4.8)	4.3 (4.1–4.6)	4.4 (4.1–4.9)	0.29	4.4 (4–4.8)	4.4 (4.2–4.9)	0.334
Aortic valve mean gradient (mmHg)	54.1 ± 17.5	52.3 ± 14.8	54.4 ± 18.1	0.609	52.9 ± 14.2	54.9 ± 19.5	0.519
Aortic regurgitation (grade II or III)	72 (57.6%)	12 (57.1%)	60 (57.6%)	0.962	33 (64.7%)	39 (52.7%)	0.182
Mitral regurgitation (grade II or III)	91 (72.8%)	15 (71.4%)	76 (73%)	0.876	40 (78.4%)	51 (68.9%)	0.24
Protetic valve Vmax (m/s)	2.2 (1.9–2.5)	2.3 (1.8–2.7)	2.2 (1.9–2.5)	0.738	2.3 (2–2.6)	2.2 (1.9–2.5)	0.537
Protetic valve mean gradient (mmHg)	11.7 ± 4.5	11.7 ± 4.4	11.7 ± 5.2	0.977	12.2 ± 4.7	11.4 ± 4.3	0.317
Paraprotetic leak	90 (72%)	14 (66.6%)	76 (73%)	0.552	34 (66.6%)	56 (75.6%)	0.272
Protetic regurgitation (grade II or III)	12 (9.6%)	2 (9.5%)	10 (9.6%)	0.989	5 (9.8%)	7 (9.4%)	0.949
**Laboratory parameters**							
Haemoglobin (g/dL)	12.4 ± 1.5	12.1 ± 1.6	12.5 ± 1.5	0.26	12.3 ± 1.6	12.5 ± 1.4	0.332
Creatinine (mg/dL)	1.05 (0.8–1.2)	1.2 (1–1.7)	1 (0.8–1.2)	**0.012**	1.1 (0.9–1.5)	0.9 (0.8–1.1)	**0.0008**
Monocyte count	587.0 ± 201.7	717.6 ± 180.7	560.6 ± 196.1	**0.001**	658.2 ± 230.2	538 ± 163.8	**0.0009**
HDL-C, mg/dL	47.8 ± 12.6	41 ± 8.9	49.2 ± 12.9	**0.006**	43.5 ± 10.8	50.7 ± 13	**0.001**
MHR	13.1 ± 5.7	18.2 ± 5.5	12.1 ± 5.2	**<0.0001**	16 ± 6.4	11.2 ± 4.2	**<0.0001**
WBC, ×10^3^/L	7.14 (6–8.1)	8 (6.8–9)	6.9 (5.8–7.9)	**0.023**	7.6 (6.7–8.9)	6.7 (5.7–7.4)	**0.0009**
Lymphocyte count, ×10^3^/L	1.6 ± 0.7	1.7 ± 0.8	1.6 ± 0.7	0.734	1.8 ± 0.8	1.5 ± 0.6	0.074
Neutrophil count, ×10^3^/L	4.9 ± 1.7	5.2 ± 1.6	4.8 ± 1.7	0.144	5.3 ± 1.7	4.6 ± 1.6	**0.025**
NLR	3.1 (2–4.2)	3.2 (2.1–4.2)	3.1 (2–4.2)	0.445	3.1 (2.2–4)	3.1 (1.9–4.2)	0.902
Fibrinogen	358.8 ± 105.5	391.9 ± 128.6	352.1 ± 99.5	0.115	386.2 ± 115.5	340 ± 94.2	**0.015**
CRP (mg/dL)	1.7 (0.9–4.5)	4.5 (1.4–13.6)	1.4 (0.9–3.2)	**0.029**	2.3 (1.1–11.2)	1.3 (0.8–2.6)	**0.015**
ESR (mm/hr)	16 (8.7–30.5)	25 (14.2–40)	15 (8–26.5)	**0.048**	20 (10–36.5)	13.5 (8–23)	**0.006**
**Type of prosthesis**							
Edwards Sapien	43 (34.4%)	8 (38%)	35 (33.6%)	0.697	21 (41.1%)	22 (29.7%)	0.187
Medtronic Evolut R	35 (28%)	7 (33.3%)	28 (26.9%)	0.552	14 (27.4%)	21 (28.3%)	0.91
ST. Jude Portico	47 (37.6%)	6 (28.5%)	41 (39.4%)	0.349	16 (31.3%)	31 (41.8%)	0.232
**Any post-procedural complication**	57 (45.6%)	7 (33.3%)	50 (48%)	0.217	23 (45%)	34 (45.9%)	0.925

Data are presented as mean ± standard deviation, median and quartiles Q1 and Q3 or n (%). CRP: C-reactive protein; ESR: erythrocyte sedimentation rate; HDL-C: high-density lipoprotein cholesterol; LVEF: left ventricular ejection fraction; MHR: monocyte-HDL-C ratio; NLR: neutrophil-lymphocyte ratio; NYHA: New York Heart Association; PSAP: pulmonary systolic arterial pressure; WBC: white blood cell. (+) means yes and (-) means no. Bold emphasises the statistical significant results.

**Table 2:** Cox regression analysis between studied parameters and overall mortality.

Parameter	Univariate Analysis		Multivariate Analysis	
	Unadjusted HR (95% CI)	*p*-Value	Adjusted HR (95% CI)	*p*-Value
MHR	1.11 (1.06–1.15)	**<0.0001**	1.1 (1.06–1.15)	**<0.0001**
Atrial fibrillation, %	2.12 (1.21–3.7)	**0.008**	1.9 (1.11–3.38)	**0.018**
Coronary artery disease	1.76 (1–3.09)	**0.048**		
Monocyte count, L	1 (1–1.003)	**0.022**		
HDL-C, mg/dL	0.95 (0.93–0.98)	**0.0006**		
WBC, ×10^3^/L	1.21 (1.06–1.38)	**0.004**		
Neutrophil count, ×10^3^/L	1.23 (1.03–1.48)	**0.02**		
Fibrinogen, mg/dL	2.12 (1.21–3.7)	0.062		
ESR, mm/h	1 (0.99–1.01)	0.146		
CRP, mg/dL	1.02 (0.98–1.05)	0.168		
Creatinine, mg/dL	1.08 (0.92–1.26)	0.326		
FEVS	0.98 (0.96–1.01)	0.255		

Regression analysis, all cases (n = 125; 51 all-cause death). CRP: C-reactive protein; ESR: erythrocyte sedimentation rate; HDL-C: high-density lipoprotein cholesterol; LVEF: left ventricular ejection fraction; MHR: monocyte-HDL-C ratio; WBC: white blood cell. Bold emphasises the statistical significant results.

## Data Availability

The data underlying this article will be shared on reasonable request to the corresponding author.
